# Long-term cost effectiveness of ticagrelor in patients with acute coronary syndromes in Thailand

**DOI:** 10.1186/s13561-014-0017-3

**Published:** 2014-11-14

**Authors:** Sukit Yamwong, Unchalee Permsuwan, Sirana Tinmanee, Piyamitr Sritara

**Affiliations:** Department of Medicine, Faculty of Medicine Ramathibodi Hospital, Mahidol University, Bangkok, 10400 Thailand; Department of Pharmaceutical Care, Faculty of Pharmacy, Chiang Mai University, Chiang Mai, 50200 Thailand; AstraZeneca (Thailand) Ltd, Bangkok, 10120 Thailand

**Keywords:** Cost-effectiveness, Ticagrelor, Acute coronary syndrome, Clopidogrel

## Abstract

**Objectives:**

To evaluate the long-term cost-effectiveness of ticagrelor and ASA versus generic and branded clopidogrel and ASA in patients with ACS based on a Thai cost database.

**Methods:**

A one-year decision tree and a long-term Markov model were constructed to estimate lifetime costs and quality-adjusted life years (QALYs). For the first year, data from PLATO (NCT00391872) were used to estimate the rate of cardiovascular events, resource use, and QALYs. For year 2 onwards, clinical effectiveness was estimated conditional on individual health states that occurred during the first year.

**Results:**

In the base-case analysis, the incremental cost-effectiveness ratio (ICER) with ticagrelor was 292,504 ($9,476) and 60,055 ($1,946) THB($)/QALY compared with generic and branded clopidogrel, respectively. The probability of ticagrelor being cost-effective was above 99% at a threshold of 160,000 THB/QALY compared with branded clopidogrel.

**Conclusions:**

This health economic analysis provides cost effectiveness data for ticagrelor compared with both generic and branded clopidogrel in Thailand. Based on this analysis, it appears that ticagrelor is an economically valuable treatment for ACS compared with branded clopidogrel within the Thai context.

**Electronic supplementary material:**

The online version of this article (doi:10.1186/s13561-014-0017-3) contains supplementary material, which is available to authorized users.

## Background

Acute coronary syndrome (ACS) is a common cardiovascular disease associated with high complication and mortality rates. From the Thai ACS Registry – a survey conducted at 17 tertiary care centers in 2007 – the in-hospital mortality rate was 12.6% [1]. A later survey in 2012 showed a lower rate of in-hospital mortality (4.8%) but the mortality rate at one-year had not decreased (17.7%) [2]. Moreover, a study of ACS patients under the Universal Coverage (UC) scheme and Civil Servant Medical Benefits Scheme (CSMBS) at all levels of hospital found that the in-hospital mortality rate was approximately 14%, and that ACS was associated with substantial health care use and costs [3,4]. Various anti-platelet drugs have been proven to reduce cardiovascular events in ACS patients. Currently, there are several classes of anti-platelet drugs available on the market including aspirin (ASA), thienopyridine products such as clopidogrel or prasugrel, and a new chemical class, the cyclopentyltriazolopyrimidines, which includes the direct-acting oral antagonist of the adenosine diphosphate P2Y_12_ receptor ticagrelor.

According to the 2012 American College of Cardiology/American Heart Association guidelines, combined treatment with ASA plus a P2Y_12_ inhibitor such as clopidogrel, prasugrel or ticagrelor is recommended as standard anti-platelet treatment in ACS [5]. However, clopidogrel is a pro-drug requiring transformation to an active metabolite, which results in a slower onset and less consistent inhibition of platelet aggregation compared with ticagrelor [6]. Ticagrelor represents a new treatment option for ACS. The PLATelet inhibition and patient Outcomes (PLATO) study was conducted to determine whether ticagrelor is superior to clopidogrel for the prevention of vascular events and death in a broad population of patients presenting with an ACS [7]. Thailand was a part of this multi-centre study. In the PLATO study, compared to clopidogrel in combination with ASA, ticagrelor in combination with ASA demonstrated superior efficacy in the prevention of thrombotic events for the composite endpoint of vascular death, myocardial infarction (MI), or stroke (9.8% vs 11.7%, hazard ratio [HR] = 0.84; 95% CI 0.77 to 0.92; p < 0.001). Trial-defined major bleeding events were similar in both treatment groups, but there were statistically significantly more combined major and minor bleeding events in the ticagrelor arm compared with the clopidogrel arm (16.1% vs 14.6%, HR = 1.11; 95% CI 1.03 to 1.30; p = 0.008) and more non-coronary artery bypass grafting (non-CABG) related major bleeding events (4.5% vs 3.8%, HR = 1.19; 95% CI 1.02 to 1.38; p = 0.03).

Treatment of ACS imposes a substantial burden on Thai society as a whole. When a new treatment becomes available, especially one with a higher cost but greater benefit than usual care, it is necessary to critically appraise the cost-effectiveness of this new treatment to determine whether the improvement in treatment efficacy makes financial sense, especially in the era of limited healthcare resources. Therefore, this study aimed to evaluate the long-term cost-effectiveness of ticagrelor and ASA versus clopidogrel and ASA in ACS patients in Thailand.

## Methods

A two-part construct model with a one-year decision tree and a Markov model developed by Nikolic et al. [8] was used to compare ticagrelor with generic and branded clopidogrel. The model was designed to capture short- and long-term costs and outcomes. The clinical effectiveness data were obtained from the PLATO trial [7] while cost data were derived from a Thai database. All costs and effects were discounted at 3% per annum as indicated by Thai guidelines [9]. Costs were presented in the year 2013 and effectiveness was measured in terms of quality adjusted-life years (QALYs). The health care payer perspective was undertaken.

### The decision model

**Figure 1:**
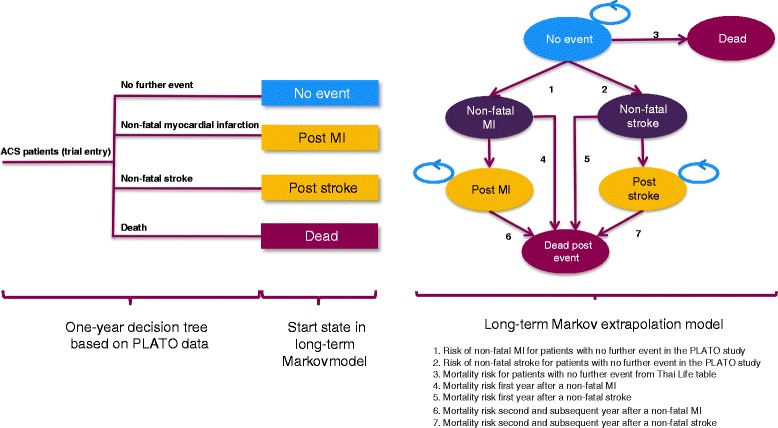
**One-year decision tree model and long-term Markov model (adapted from PLATO) [** 7 **].**

To model the short-term cost-effectiveness of ticagrelor, a one-year decision tree covering four mutually exclusive health states (no further event, non-fatal MI, non-fatal stroke, and death from any cause) was constructed as shown in Figure 1. During this phase, each ACS patient received clopidogrel 75 mg once daily plus ASA 75–100 mg or ticagrelor 90 mg twice daily plus ASA 75–100 mg for 12 months. We assumed the treatment duration lasted only one year and that there was no rebound effect for treatment remaining in the Markov cycles. At the end of one year, patients were allocated to one of the six mutually exclusive health states in the Markov model: no further event, non-fatal MI, post MI, non-fatal stroke, post stroke and dead (Figure 1). The non-fatal MI and non-fatal stroke health states were tunnel states allowing for a worse prognosis for patients in the year in which a non-fatal event occurred compared to a subsequent year. Patients in the post-MI or post-stroke states would remain in those states for all succeeding cycles until they moved to death state. The cycle length was one year with a lifetime horizon. We assumed our Thai cohort of ACS patients had a median age of 62 years (based on PLATO data [7] and consistent with the average age of ACS patients in the Thai Registry report [2]) and had similar percentages of ST segment elevation myocardial infarction (STEMI), non-ST segment elevation myocardial infarction (NSTEMI), and unstable angina (UA) as patients enrolled in the PLATO trial (Table 1) [7].

**Table 1:** Baseline characteristics of patients in the PLATO study, by treatment group* [7]

**Characteristic**	**Ticagrelor group (N = 9333)**	**Clopidogrel group (N = 9291)**
Age — median (range)	62.0 (19–97)	62.0 (21–94)
Female sex — no. (%)	2655 (28.4)	2633 (28.3)
BMI — median (range)	27 (13–68)	27 (13–70)
Cardiovascular risk factor — no./total no. (%)		
Habitual smoker	3360 (36.0)	3318 (35.7)
Hypertension	6139 (65.8)	6044 (65.1)
Dyslipidemia	4347 (46.6)	4342 (46.7)
Diabetes mellitus	2326 (24.9)	2336 (25.1)
Other medical history — no./total no. (%)		
MI	1900 (20.4)	1924 (20.7)
Percutaneous coronary intervention	1272 (13.6)	1220 (13.1)
Congestive heart failure	513 (5.5)	537 (5.8)
Final diagnosis of ACS — no./total no. (%)		
ST-elevation MI	3496 (37.5)	3530 (38.0)
Non–ST-elevation MI	4005 (42.9)	3950 (42.5)
Unstable angina	1549 (16.6)	1563 (16.8)
Other diagnosis or missing data	283 (3.0)	248 (2.7)

*Adapted from PLATO [7].

### Effectiveness parameters

For a one-year decision tree, the effectiveness data were derived exclusively from the PLATO trial [7], as Thailand was included in this multi-centre study. A parametric time-to-event survival model with a Weibull distribution was employed in order to determine the baseline risk of events with clopidogrel treatment and a hazard ratio (HR) of ticagrelor treatment effect. Hence, the ticagrelor event rates were the product of baseline risk and a ticagrelor HR (Table 2).

**Table 2:** **Input parameters and values used in the economic model**

**Input parameters**	**Value**	**Source**
**One-year decision tree**		
*** Ticagrelor***		
No event	0.894	PLATO [7], Nikolic [8]
Non-fatal MI	0.050	PLATO [7], Nikolic [8]
Non-fatal stroke	0.010	PLATO [7], Nikolic [8]
Death any cause	0.046	PLATO [7], Nikolic [8]
*** Clopidogrel***		
No event	0.875	PLATO [7], Nikolic [8]
Non-fatal MI	0.057	PLATO [7], Nikolic [8]
Non-fatal stroke	0.009	PLATO [7], Nikolic [8]
Death any cause	0.059	PLATO [7], Nikolic [8]
** Markov model** ^a^		
Annual risk of MI in the no event state	0.019	PLATO [7], Nikolic [8]
Annual risk of stroke in the no event state	0.003	PLATO [7], Nikolic [8]
Risk of death in no event state^b^	2.000	Thai Life table [10], Allen [11], Taneja [12]
Risk of death in the non-fatal MI state^b^	6.000	PLATO [7], Nikolic [8]
Risk of death in the post MI state^b^	3.000	PLATO [7], Nikolic [8]
Risk of death in the non-fatal stroke state^b^	7.430	Dennis [13], Hanke [14]
Risk of death in the post stroke state^b^	3.000	PLATO [7], Nikolic [8]

^a^The values in the Markov model are the same for ticagelor and clopidgrel.

^b^Hazard ratio over standard mortality.

Due to patients no longer being on study medications after one year according to our assumption, the identical transition probabilities for the second year onwards were employed in both treatment strategies in the Markov model. The difference in both groups occurred for the proportion of patients in the different start states in the Markov model. The transition probabilities from no event to non-fatal MI or non-fatal stroke in the Markov model were estimated with an extrapolation of the observed HRs in the clopidogrel arm of PLATO beyond 1 year [7,8]. The annual mortality rate (MR) in the no-event state was estimated using age- and gender-specific MR from Thai lifetables [10]. To incorporate an increased risk of mortality associated with ACS, a HR was applied to the age- and gender-specific MR of the Thai population. Patients suffering an ACS event have a relatively high risk of a fatal event occurring within one year and the risk declines over time the longer that patients survive without a subsequent ACS event [11,12]; hence, the risk of death for patients in the no-event state was double that of standard mortality risk from Thai lifetables. The mortality risks of non-fatal MI, post-MI, and post-stroke were estimated by extrapolation of PLATO trial data [7]. The mortality risks of non-fatal stroke were derived from the literature [13,14].

QALY is the product of utility and life-years gained. Utilities within the one-year decision tree were based on EQ-5D data collected within the PLATO study [7,8]. In the Markov model, the baseline utility of the no-event state was also derived from the PLATO trial [7,8]. A decrement due to each health state was applied based on a previous study conducted in Thailand [15] (Table 3).

**Table 3:** **Base-case utility values**

**Parameters**	**Value**	**Sources**
**One-year decision tree**		
*** Ticagrelor***		
No event	0.873	PLATO [7], Nikolic [8]
Non-fatal MI	0.811	PLATO [7], Nikolic [8]
Non-fatal stroke	0.735	PLATO [7], Nikolic [8]
Death any cause	0.247	PLATO [7], Nikolic [8]
*** Clopidogrel***		
No event	0.876	PLATO [7], Nikolic [8]
Non-fatal MI	0.814	PLATO [7], Nikolic [8]
Non-fatal stroke	0.738	PLATO [7], Nikolic [8]
Death any cause	0.250	PLATO [7], Nikolic [8]
** Markov model** ^a^		
No event aged less than 70 years	0.875	PLATO [7], Nikolic [8]
No event aged 70–79 years	0.843	PLATO [7], Nikolic [8]
No event aged over 79 years	0.781	PLATO [7], Nikolic [8]
Annual utility decrement MI 1^st^ year Markov model and 2^nd^ year onward	0.147	Tamteeranon [15]
Annual utility decrement stroke 1^st^ year Markov model and 2^nd^ year onward	0.226	Tamteeranon [15]

^a^The values in the Markov model are the same for ticagelor and clopidogrel.

### Resource and cost parameters

This study was carried out from the viewpoint of the payer; hence, only direct medical costs were taken into consideration. These included study medication, concomitant medications, hospitalizations, investigations, interventions, and bleeding-related resources. For the one-year decision tree, healthcare costs incurred in each treatment arm were based on resource used data from the PLATO trial [7,8] and unit costs of those resources from data in Thailand (Additional file 1: TableS1) [16,17]. The cost of ticagrelor used was based on the manufacturer’s price (94.16 THB per day). Clopidogrel costs were derived from the Drug and Medical Supply Information Center (DMSIC) in Thailand (branded = 72.53 THB per day and the cheapest generic = 0.94 THB per day) [18]. The drug costs included 7% VAT. For year 2 onwards, the individual total cost of each health state was exclusively based on data from Thailand [4,15]. All costs were adjusted to the year 2013 using the consumer price index (medical care component) [19]. Table 4 presents the total costs used in this study.

**Table 4:** **Total costs used in the model** ^**a**^

**Items**	**Cost (THB)**	**Sources**
**One-year decision tree**		
*** Ticagrelor***		
Annual drug cost	34,368	Manufacturer’s price
No event	117,404	Nikolic [8], Riewpaiboon [16]
Non-fatal MI	204,851	Nikolic [8], Riewpaiboon [16], Central office for Healthcare Information [17]
Non-fatal stroke	141,918	Nikolic [8], Riewpaiboon [16]
Death any cause	120,132	Nikolic [8], Riewpaiboon [16]
*** Clopidogrel***		
Annual drug cost (branded price)	26,473	DMSIC [17]
Annual drug cost (generic price)	344	DMSIC [17]
No event	120,307	Nikolic [8], Riewpaiboon [16]
Non-fatal MI	207,753	Nikolic [8], Riewpaiboon [16], Central office for Healthcare Information [17]
Non-fatal stroke	144,821	Nikolic [8], Riewpaiboon [16]
Death any cause	123,034	Nikolic [8], Riewpaiboon [16]
** Markov model**		
Total cost MI 1^st^ year Markov model	168,196	Anukoolsawat [4]
Total cost MI 2^nd^ year and onward	35,926	Anukoolsawat [4]
Total cost stroke 1^st^ year Markov model	79,800	Tamteeranon [15]
Total cost stroke 2^nd^ year and onward	12,642	Tamteeranon [15]
Total cost of no event	21,866	Anukoolsawat [4]

^a^Detailed table of Thai hospital charges and unit cost (Baht) for the resource use collected in the PLATO trial provided in the Additional file 1: Table S1.

### Model analyses

Costs and QALY were calculated over a lifetime horizon and presented as the incremental cost-effectiveness ratio (ICER) which was the ratio of an incremental cost and an incremental QALY. The model was constructed and run using Microsoft Excel version 2007.

### Sensitivity analyses

A series of sensitivity analyses were carried out to address uncertainty of parameters in the model. One-way sensitivity analysis was conducted to assess the uncertainty surrounding each parameter individually. The variables tested in the one-way sensitivity analysis included all transition probabilities, utilities and cost parameters. Transition probabilities were varied first by ±20%, and then by a reduction of 50% and an increase of 200%. All cost parameters were varied by 20% except drug costs, the ticagrelor cost was varied by 10% while the cost of the least expensive generic clopidogrel was set at 10% lower than current prices, and the cost of branded clopidogrel was set at 10% higher than current price. The results were presented as a tornado diagram. Probabilistic sensitivity analysis (PSA) was also undertaken. With the assigned distribution to each model parameter, uncertainty was then propagated through the model using Monte Carlo Simulation with parameter values drawn at random. The decision analysis model was simulated on a thousand iterations. The PSA results of ticagrelor vs branded clopidogrel and ticagrelor vs generic clopidogrel were presented as cost-effectiveness acceptability curves. For the Thai context, an ICER of less than 160,000 THB/QALY is considered cost effective [20]. This is consistent with WHO recommendations that an ICER of 1GDP per capita demonstrates acceptable cost-effectiveness, while an ICER of over 3GDP per capita is justified to be considered not cost-effective [21]. The GDP per capita in Thailand in 2013 was 174,658 THB [22,23].

## Results

The results showed that, compared with either generic or branded clopidogrel, ticagrelor treatment had a higher total cost with higher QALY. However, the difference in cost was less marked in analyses using branded versus generic clopidogrel (6,553 vs 31,918 THB respectively) resulting in a lower ICER of branded clopidogrel than generic clopidogrel (60,055 vs 292,504 THB/QALY respectively) compared with ticagrelor (Table 5).

**Table 5:** **Base-case result**

	**Ticagrelor**	**Clopidogrel**	**Incremental**	**ICER**
**Ticagrelor vs generic clopidogrel**				
Costs (THB)	368,747	336,829	31,918	
Life-years	9.201	9.079	0.122	261,197
QALYs	7.711	7.602	0.109	292,504
**Ticagrelor vs branded clopidogrel**				
Costs (THB)	368,747	362,194	6,553	
Life-years	9.201	9.079	0.122	53,627
QALYs	7.711	7.602	0.109	60,055

QALYs: Quality adjusted-life years; ICER: Incremental cost-effectiveness ratio.

### Sensitivity analysis

**Figure 2:**
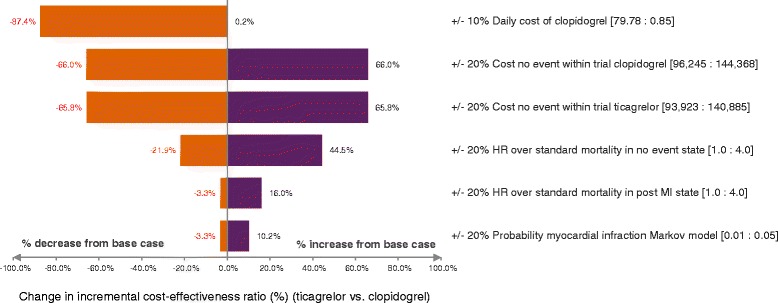
**Tornado diagram for one-way sensitivity analysis of ticagrelor vs. clopidogrel.**

**Figure 3:**
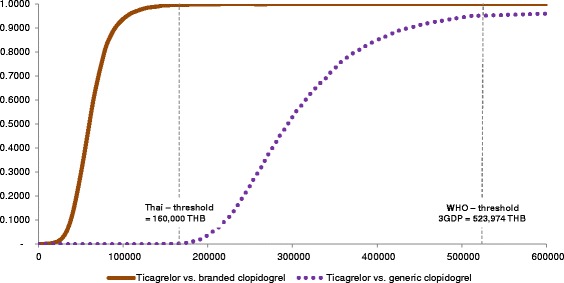
**Cost-effectiveness acceptability curves.**

Of the 26 variables tested in one-way sensitivity analyses, we found that the cost of clopidogrel, the cost of the no-event state within the trial of both ticagrelor and clopidogrel, and the HR of standard mortality in the no-event state had the greatest impact on ICER (Figure 2). Compared with branded clopidogrel, ticagrelor had more than 99% probability of being cost-effective within the predefined threshold of 160,000 THB/QALY. However, ticagrelor was less cost-effective compared with generic clopidogrel (Figure 3).

## Discussion

We conducted a cost-effectiveness study using an analytical model to examine whether the new treatment, ticagrelor in combination with ASA, was cost-effective compared with either generic or branded clopidogrel in combination with ASA for treating ACS patients from a healthcare payer perspective. Our model was composed of a one-year decision tree and a Markov model to capture short- and long-term outcomes. We found that the price of clopidogrel had the greatest impact on ICER. Compared with generic clopidogrel (0.94 THB per day), ticagrelor had a cost per QALY gained of 292,504 THB which was greater than the threshold of 160,000 THB/QALY. However, when branded clopidogrel was used as the comparator, ticagrelor treatment was cost-effective within the predefined threshold for Thailand.

This study is predicated on the assumption that generic clopidogrel is equally as efficacious and safe as the branded counterpart. However, Gomez et al. reported that most clopidogrel copies are not of equivalent quality [24]. A high number of impurities were found in many copies; over 60% of the generic copies contained more than four times the amount of hydrolysis product or R-enantiomer compared with branded clopidogrel. In addition, 50% of the samples did not comply with the 95–105% limits for content. As a consequence, the results of ACS treatment with generic clopidogrel might not be the same as branded clopidogrel, especially in a very high-risk group, which would affect the conclusions of our analysis.

Several limitations of this study need to be mentioned. Firstly, the information on short-term resource use for the treatment of ACS was based on data from the PLATO trial [7], which was conducted in accordance with a strict protocol, and may not accurately reflect real practice in ACS management in Thailand. We reconciled this overestimated cost by using the unit cost item from Thai data.

Secondly, due to a paucity of data from Thailand, we estimated the cost of the no-event state in the Markov model from outpatient visit and investigation costs from the 1-year cost of MI reported in Anukoolsawat [4], but excluding inpatient and intervention costs. As a result, the no-event total cost was higher than the total cost of 2^nd^ year stroke in the Markov model. This limitation was due to the fact that available published reports on the cost of MI and stroke were based on data collected from two different centers, a medical school (for MI) and a government neurological institute (for stroke). The costs might not be comparable because of different treatment and resource use patterns at different centers. To address this, we tested the impact of stroke cost parameters on the model results in the sensitivity analyses. We varied the cost of stroke during the first year and from year 2 onwards in the Markov model by 100% and found that the ICER results were not sensitive to these two parameters.

A third limitation arises from the structure of the Markov model, which does not explicitly allow patients to suffer multiple cardiovascular events in their lifetime. Furthermore, this study is based on a payer perspective and limited only to direct medical costs. Direct non-medical costs such as transportation or indirect costs such as lost productivity are not taken into consideration. The finding from Anukoolsawat [4] reported that ACS was associated with a high economic burden for patients and their families resulting from loss of productivity, and that the indirect cost of ACS was even higher than the direct health care costs. Therefore, excluding these types of costs underestimates the true total cost of ACS to Thai society.

## Conclusions

This health economic analysis supports the cost effectiveness of ticagrelor compared with branded clopidogrel in Thailand. The ICER of ticagrelor compared with branded clopidogrel is 60,055 THB/QALY. Therefore, ticagrelor appears to be an economically valuable treatment for ACS compared with branded clopidogrel within the Thai context.

## Additional file

Additional file 1: Table S1. Costs used in the model.
