# Supplementary material for: Long-term cost effectiveness of ticagrelor in patients with acute coronary syndromes in Thailand
Source: Health Econ Rev. 2014 Nov 14;4:17. doi: 10.1186/s13561-014-0017-3 (PMC4502070; doi:10.1186/s13561-014-0017-3)
Supplement: Additional file 1: Table S1. — Costs used in the model. [file 13561_2014_17_MOESM1_ESM.doc]

**Additional file 1: Table S**1. Costs used in the model

| **Items** | **Cost (THB)** | **Sources** |
| --- | --- | --- |
| *Hospitalizations* |  |  |
| Bed day cardiology ward | 1,378 | Riewpaiboon [16] |
| Bed day general ward | 1,378 | Riewpaiboon [16] |
| Bed day coronary care unit | 2,636 | Riewpaiboon [16] |
| Bed day thoracic intensive care unit | 2,636 | Riewpaiboon [16] |
| Bed day intensive care unit | 2,636 | Riewpaiboon [16] |
| *Investigations* |  |  |
| Stress test | 2,039 | Riewpaiboon [16] |
| Echocardiography | 4,079 | Riewpaiboon [16] |
| Myocardial scintigraphy | 12,000 | Adapted from Central office for Healthcare Information [17] |
| Electrophysiology study | 50,000 | Adapted from Central office for Healthcare Information [17] |
| Holter study | 2,719 | Riewpaiboon [16] |
| Ventilation/perfusion scan | 5,438 | Riewpaiboon [16] |
| Pulmonary angiography | 15,000 | Adapted from Central office for Healthcare Information [17] |
| Coronary angiography | 20,393 | Riewpaiboon [16] |
| Computer tomography |  |  |
| Head/brain | 5,235 | Riewpaiboon [16] |
| Spinal | 7,274 | Riewpaiboon [16] |
| Chest | 6,798 | Riewpaiboon [16] |
| Helical | 20,000 | Adapted from Central office for Healthcare Information [17] |
| Abdomen | 9,857 | Riewpaiboon [16] |
| Extremity | 6,798 | Riewpaiboon [16] |
| Magnetic resonance imaging |  |  |
| Head/brain | 10,876 | Riewpaiboon [16] |
| Spinal | 10,876 | Riewpaiboon [16] |
| Chest | 10,876 | Riewpaiboon [16] |
| Abdomen | 16,314 | Riewpaiboon [16] |
| Extremity | 10,876 | Riewpaiboon [16] |
| *Interventions* |  |  |
| Pacemaker | 13,595 | Riewpaiboon [16] |
| Implantable cardiac defibrillator | 310,000 | Adapted from Central office for Healthcare Information [17] |
| Intra-aortic balloon pump | 36,400 | Adapted from Central office for Healthcare Information [17] |
| Percutaneous coronary intervention without stent | 40,273 | Adapted from Central office for Healthcare Information [17] |
| Percutaneous coronary intervention with stent (excl. stent cost) | 67,978 | Riewpaiboon [16] |
| Bare metal stent | 6,800 | Central office for Healthcare Information [17] |
| Drug eluting stent | 30,000 | Central office for Healthcare Information [17] |
| Coronary artery bypass grafting |  |  |
| Without valve replacement | 33,882 | Riewpaiboon [16] |
| With valve replacement | 35,242 | Riewpaiboon [16] |
| *Bleeding related* |  |  |
| Reoperation due to bleedings | 30,000 | Adapted from Central office for Healthcare Information [17] |
| Units of packed red blood cells | 721 | Riewpaiboon [16] |
| Units of whole blood | 966 | Riewpaiboon [16] |
| Units of fresh frozen plasma | 523 | Riewpaiboon [16] |
| Units of platelets | 530 | Riewpaiboon [16] |
